# Conservative Rehabilitation Provides Superior Clinical Results Compared to Early Aggressive Rehabilitation for Rotator Cuff Repair: A Retrospective Comparative Study

**DOI:** 10.3390/medicina55080402

**Published:** 2019-07-24

**Authors:** Umile Giuseppe Longo, Giacomo Rizzello, Stefano Petrillo, Mattia Loppini, Nicola Maffulli, Vincenzo Denaro

**Affiliations:** 1 Department of Orthopaedic and Trauma Surgery, Campus Bio-Medico University, 00128 Rome, Italy; 2 Department of Biomedical Sciences, Humanitas University, Via Rita Levi Montalcini 4, 20090 Pieve Emanuele, Milan, Italy; 3 Department of Orthopaedic and Trauma Surgery, Humanitas Clinical and Research Center, Via Alessandro Manzoni 56, 20089 Rozzano, Milan, Italy; 4 Centre for Sports and Exercise Medicine, Barts and The London School of Medicine and Dentistry, Mile End Hospital, London E1 4DG, UK

**Keywords:** rotator cuff, arthroscopy, rehabilitation, outcomes, conservative protocol, accelerated protocol, rotator cuff repair

## Abstract

*Background and objectives:* To compare the long term clinical outcomes, range of motion (ROM) and strength of two different postoperative rehabilitation protocols after arthroscopic rotator cuff repair (RCR) for full-thickness rotator cuff (RC) tears. *Materials and Methods:* Patients undergoing RCR were divided into two groups. In 51 patients (56 shoulders), rehabilitation was performed without passive external rotation, anterior elevation ROM, and active pendulum exercises in the first 2 weeks after surgery (Group A). In 49 patients (50 shoulders) aggressive rehabilitation was implemented, with early free passive external rotation, anterior elevation ROM, and active pendulum exercises were allowed from the day after surgery (Group A). *Results:* No statistically significant differences were found in clinical scores, muscle strength, passive forward flexion, passive and active internal/external rotation between the two groups. However, the mean active forward flexion was 167.3° ± 26° (range 90–180°) in group A and 156.5° ± 30.5° (range 90–180°) in group B (*p* = 0.04). *Conclusions:* A statistically significant difference between the 2 groups was found in active forward flexion ROM, which was better in patients of group A.

## 1. Introduction

Lesions of the rotator cuff (RC) are common [1], especially in middle age individuals [2,3,4,5,6,7], but may also occur in young active patients [1,8,9,10,11]. They produce pain and loss of function of the affected arm, being one of the major causes of reduced work activities, with decreased health-related quality of life [12,13,14,15] and representing a high cost for National Health Systems [7,16,17].

The management of full-thickness RC tears is often surgical. Various arthroscopic operative techniques allow good clinical and functional outcomes. Despite advances in arthroscopic techniques [18,19,20,21], high rates of functional and structural failure after arthroscopic RCR have been reported [22,23].

Various surgical and nonsurgical factors may affect whether a tendon will successfully heal after repair [8,9,10,20,24,25,26,27]. Nonsurgical factors include size and chronicity of the tear, muscle atrophy or fatty degeneration, and patients’ age. On the other hand, surgical factors include repair technique and postoperative rehabilitation [8,11].

Early rehabilitation plays a critical role to obtain good results after RC repair. This is recommended for preventing postoperative stiffness but can be considered one of the factors that may affect clinical, functional and structural outcomes [28,29,30,31,32]. Indeed, pain, strength, function, and range of motion (ROM) significantly improve after arthroscopic RC repair, regardless of postoperative rehabilitation protocols, but aggressive early motion may increase anatomic failure of the repaired RC [33].

Only a few studies have compared clinical and functional outcomes, as well as strength, of two different early rehabilitation protocols after rotator cuff repair (RCR) for full-thickness RC tear.

The purpose of the present investigation was to compare the long term clinical outcomes, ROM and strength between two different early rehabilitation protocols after arthroscopic RCR for full-thickness RC tears.

## 2. Materials and Methods

All participants were recruited at the University Hospital Campus Bio-Medico of Rome. All subjects gave their informed consent for inclusion before they participated in the study. The study was conducted in accordance with the Declaration of Helsinki, and the protocol was approved by the Ethics Committee of Campus Bio Medico of Rome (Prot 37/16 OSS ComET CBM date 14-9-2016). Authors declare that the investigations were carried out following the rules of the Declaration of Helsinki of 1975, revised in 2013.

### 2.1. Eligibility Criteria

Patients were included in the study if they presented the following conditions at the time of surgery: RC tears diagnosed on clinical grounds; no episodes of shoulder instability; no radiographic signs of fracture of the glenoid or the greater or lesser tuberosity; magnetic resonance imaging (MRI) evidence of RC tear; duration of symptoms of at least three months; inadequate response to non-operative management (including non-steroidal anti-inflammatory drugs, physiotherapy, rest, and one local corticosteroid injection). Patients with partial or massive RC tears were excluded, while patients with biceps lesions or tendinopathy were included in the study.

Patients were excluded if they presented the following conditions at the time of surgery: inflammatory joint disease; prior surgery on the affected shoulder; labral pathology amenable to surgical repair; degenerative arthritis of the glenohumeral joint; symptomatic arthritis of the acromioclavicular joint; RC arthropathy.

At the time of the last follow up, patients were also excluded if unable to complete questionnaires because of language problems or cognitive disorders.

Materials and Methods should be described with sufficient details to allow others to replicate and build on published results. Please note that publication of your manuscript implicates that you must make all materials, data, computer code, and protocols associated with the publication available to readers. Please disclose at the submission stage any restrictions on the availability of materials or information. New methods and protocols should be described in detail while well-established methods can be briefly described and appropriately cited.

Research manuscripts reporting large datasets that are deposited in a publicly available database should specify where the data have been deposited and provide the relevant accession numbers. If the accession numbers have not yet been obtained at the time of submission, please state that they will be provided during review. They must be provided prior to publication.

Interventional studies involving animals or humans, and other studies require ethical approval and must list the authority that provided approval and the corresponding ethical approval code.

### 2.2. Patient Recruitment

100 patients (106 shoulders) followed two types of postoperative rehabilitation after arthroscopic RC repair. 49 patients (50 shoulders) underwent limited rehabilitation, in which early free passive external rotation, active elevation ROM, and active pendulum exercises were restricted (Group A), while in the remaining 51 patients (56 shoulders) the rehabilitation protocol performed included early passive free ROM and pendulum exercises (Group B). For all 100 patients assigned to one of the two treatments, an average of 4 ± 1-year results were available.

### 2.3. Preoperative Evaluation

At the time of surgery, all patients underwent MRI scans and oblique coronal, oblique sagittal and axial T2-weighted spin-echo MRIs (repetition time (RT): 3200 ms; echo time (ET): 85 ms). Furthermore, a pre-operative assessment using standard radiographs (anteroposterior projections, neutral, external and internal rotation, lateral view of the scapula, and axillary view) was performed in all patients.

### 2.4. Postoperative Evaluation

Two orthopedic surgeons blindly performed all the measurements. All patients were evaluated for age; sex; arm dominance; history of trauma; location of the RC tear; dimension of the RC tear; biceps tendon lesion or tendinopathy; type of treatment of biceps tendon; number of suture anchors used for RC repair.

A modified UCLA [34] shoulder rating scale was used to evaluate postoperative shoulder pain (10 points), function (10 points), active forward flexion (5 points), strength (5 points) and patient satisfaction (5 points). The maximum score obtainable is 35, and the results were classified as excellent (34–35 points), good (28–33), fair (21–27), or poor (0–20). The Wolfgang criteria [35] were used to assess postoperative shoulder pain (4 points), active abduction (4 points), strength (4 points) and patient satisfaction (1 point or minus 1 point). The maximum score obtainable is 17, and the results were classified as excellent (14–17 points), good (11–13 points), fair (8–10 points) or poor (0–7 points). All patients postoperatively completed the Italian version of the Oxford Shoulder Score (OSS) [36,37], a self-administered 12 item questionnaire that evaluates shoulder function, pain and strength in relation with daily life activities. The minimum score is 12 points, and the maximum is 60 points. The higher the score, the worse the condition of the shoulder [38].

The assessment of postoperative ROM was performed using a standard universal goniometer with the scale marked in one-degree increments. The measurements of supine passive forward elevation (sagittal plane), passive internal and external rotation (90° abduction) were obtained using standard measurement [39]. Patients were positioned supine on an examining couch with the shoulder at 90° of abduction in the scapular plane (approximately 15° anterior to the coronal plane). Care was taken to fix the scapula with one hand while the other hand of the examiner rotated the shoulder into position [40]. One orthopedic surgeon held the shoulder position, while a second orthopedic surgeon obtained the measurement after a firm endpoint was established. The forearm was held in neutral rotation during rotational measurement. The assessments of active anterior elevation, active external and internal rotation degrees were also obtained using standard measurement guidelines [41]. The assessment of the strength during anterior elevation, internal and external rotation was performed using a dynamometer (model 01163, Lafayette Instrument Company, Lafayette, Indiana). The results obtained were expressed in Newton (N). Both examiners performed three measurements for each ROM and strength measurement investigated. The average value for each variable was used for statistical purposes.

### 2.5. Sample Size and Demographic Details

The mean follow up was 4 + 1 years ranging from 1–10 years. There were 46 male and 54 female, with a mean age of 56.9 + 9.4 (range 29–76 years).

Demographic and surgical details of the patients enrolled in the study are shown in Table 1.

### 2.6. Arthroscopic Technique

Patients underwent brachial plexus block (associated, in selected patients, with general anesthesia), and were placed in a lateral decubitus position. The arm was suspended at approximately 45° of abduction and 20° of forward flexion. The distraction of the shoulder joint was accomplished with 4.5 to 6.5 kg of traction. Diagnostic arthroscopy was then performed to evaluate the extent of the RC tear, any lesions of the biceps tendon, and other associated lesions. To control bleeding, we used radiofrequency, adrenalin admixture to the irrigation fluid, and asked the anesthesiologist to lower the systolic blood pressure to 90 mm Hg, if possible. An arthroscopic pump maintained fluid pressure at 40 mmHg, increasing it temporarily on demand. A subacromial decompression, consisting of the release of coracoacromial ligament, anteroinferior acromioplasty, and subacromial bursectomy was performed in the presence of a type III acromion, acromial spurs or according to surgeon decision. The lateral portal was used to mobilize the RC back to its bony insertion. Using a burr through the lateral portal, the footprint of the greater tuberosity was abraded. The RCR was performed placing one or two rows of suture anchors double-loaded just in the lateral aspect of the footprint. The number of suture anchors and of suture rows varied with the size of the tear, the type of repair techniques and the surgeon decision. At the end of surgical procedure, a drain was left in situ in all patients, and removed 24 h after surgery.

### 2.7. Post-Operative Management

The postoperative rehabilitation regimen was different between the two groups.

Patients in group A (early passive external rotation and active pendulum exercises not allowed) performed passive assisted shoulder stretching exercises with limitation of ROM. Passive exercises allowed for the first 2 weeks from the operation included only passive forward flexion up to a tolerable range, while passive external rotation exercises and active pendulum exercises were restricted. On the other hand, active elbow flexion and extension were allowed. Patients were instructed to perform exercises 5 times a day, with 10 repetitions of each movement each time. Patients were allowed to do this at home, only when they demonstrated to the authors complete autonomy in exercise execution. Exercises were performed by patients at prearranged hours (8.00 am, 11.00 am, 2.00 pm, 5.00 pm, and 8.00 pm). Each session lasted about 30 min. The rest time was 1 minute. The arm was supported during rest, using a sling with an abduction pillow for 6 weeks. After 2 weeks from the operation, passive external rotation up to 30° ROM and pendulum exercises were introduced. Active assisted exercises and muscle strengthening exercises were introduced after 6 weeks from the operation according to a validated postoperative protocol (http://www.moonshoulder.com/impactstudy.html). Isoinertial strengthening and rehabilitation of the RC, deltoid and scapular stabilizers were initiated 10–12 weeks after the operation. Sports activities, heavy manual work, and overhead activities were allowed after the OSS became >20, which occurred 6 months after surgery.

Patients in group B (early passive external rotation exercises and active pendulum exercises allowed) followed an aggressive assisted rehabilitation protocol. They had the same protocol of group A except for passive forward flexion, active pendulum exercises, and passive external rotation up to 30° that were allowed from the day after surgery. Overhead stretching was restricted until 6 weeks post-operatively. Isoinertial strengthening and rehabilitation of the RC, deltoid and scapular stabilizers were initiated 10–12 weeks after the operation, as previously described.

Rehabilitation was continued for 6 months in both groups according to a validated postoperative protocol (http://www.moonshoulder.com/impactstudy.html).

### 2.8. Statistical Analysis

All the statistical evaluations were conducted using the 19.0 version of SPSS (Chicago, IL, USA) for Mac.

Statistical analysis was performed considering the following outcomes: total modified UCLA shoulder score; total Wolfgang criteria shoulder score; total OSS. We considered also passive and active ROM, strength of anterior elevation, internal rotation, and external rotation ROM. We analyzed the following independent variables: age; sex; arm dominance; history of trauma; dimension of the RC tear; biceps tendon rupture or tendinopathy; type of treatment of biceps tendon; number of anchors used for the RC repair. Comparison between the two groups for each independent variable was carried out with the Student’s *t-*test for continuous variables and the *x*2 test for categorical variables. Significance was set at *p* < 0.05, and the confidence level was 95%.

## 3. Results

No patients reported postoperative complications. We report clinical and functional results at an average follow up of 4 ± 1 years (range: 1–10 years).

### 3.1. Clinical Scores

No statistically significant differences were found in clinical scores between the two groups (Table 2).

### 3.2. ROM and Strength

No statistically significant differences were found in passive forward flexion, passive and active internal/external rotation between the two groups. Moreover, no differences statistically significant were found in muscle strength between the two groups. However, the mean active forward flexion was 167.3° ± 26° (range 90–180°) in group A and 156.5° ± 30.5° (range 90–180°) in group B (*p* = 0.04), resulting statistically significant (Table 3).

## 4. Discussion

Rehabilitation plays a critical role to obtain satisfactory outcomes after arthroscopic RC repair and is considered as crucial as surgery for successful healing of the injured tendons. The joint motion produced during rehabilitation exercises affects RC healing because it imparts stresses on the repaired RC tendons, which may lead to failure. Gerber et al. [42] demonstrated in a sheep model that no repair is able to withstand the high loads imposed by weight-bearing. Park et al. [43] showed in a biomechanical study that external rotation ROM after RCR can produce gap formation in the anterior portion of the supraspinatus tendon. Bigliani et al. [44] demonstrate that 16% of failed RCR were related to postoperative rehabilitation, while Cummins et al. [45] found that the most common cause of failure in patients undergoing revision RCR was suture pulling through the repaired tendon.

Lee et al. [34] conducted a study in which two different rehabilitation protocols after RCR were compared. They demonstrated that an aggressive early rehabilitation protocol after RCR is associated with better ROM until 3 months when compared with a limited early passive motion rehabilitation protocol. However, the retear rate of the aggressive early passive motion rehabilitation protocol was more than twice the rate of the limited early passive motion rehabilitation group. After 6 months from the RC repair, no differences were present concerning ROM between the two study groups, except for internal rotation at 90° of abduction. Moreover, none of the differences between the 2 groups regarding ROM and retear rate was statistically significant. Cuff et al. [46] performed a randomized controlled trial to understand whether a delayed physical therapy protocol, which limits the early passive range of shoulder motion produced different results from early postoperative physical therapy protocol after arthroscopic RC repair. They concluded that there are no significant differences in patient satisfaction, RC healing, or ROM between the early and delayed groups.

In our study, we compared two different rehabilitation protocols after RCR for the management of full-thickness RC tears. The first rehabilitation protocol was a limited rehabilitation protocol in which passive external rotation and anterior elevation ROM, as well as active pendulum exercises, was restricted. The second protocol was a more aggressive rehabilitation protocol, in which both passive forward flexion and passive external rotation up to 30° ROM, as well as active pendulum exercises, were allowed from the day after surgery. In the present study, at a mean follow up of 4 years, a statistically significant difference was found concerning anterior elevation ROM between the 2 groups. Better results were obtained by the patients who received a limited early passive motion rehabilitation protocol. Probably the early aggressive rehabilitation protocol determines a retear rate higher than the early limited rehabilitation protocol, validating the results of our study. Moreover, in the case of subscapularis repair, passive external rotation ROM should be restricted to avoid damage to the repair. Other differences between the two groups in muscle strength, active and passive ROM and clinical scores (UCLA, Wolfgang, and OSS) were not statistically significant.

Our study was conducted on 2 groups of patients whose characteristics were homogeneous for all variables considered, confirming that the sample chosen for the study was appropriate. We decided to include only patients with small, medium or large RC tears, excluding patients with massive RC tears, because we believe that clinical and functional outcomes in patients with massive RC tears should be considered separately. Moreover, our selection and recruitment process, our assessment criteria and our follow up were extremely rigorous and performed in a strict scientific fashion. With the number of patients enrolled, the results of our study can be considered univocal.

Major strengths of the present study are: (1) two fully trained surgeon (G.R. and F.F.), each with over 25 years of experience, performed all the operation using a well-defined established technique; (2) no patients experienced surgical complications; (3) the follow up evaluations were performed blindly by two independent examiners (U.G.L and S.P.); (4) the evaluation of ROM and strength was performed according to standard measurement guidelines; (5) the mean follow up is long enough to consider that, by then, the results of surgery had stabilized and recovery effected; (6) the number of patients enrolled in the study can guarantee adequate results; (7) patients with massive RC tears were excluded; (8) all the patients were followed by a full-trained physiotherapist during the rehabilitation exercises.

However, several limitations of our study must be underlined: (1) we did not obtain a base-line evaluation and a base-line clinical score; (2) we included patients with an RC tear regardless of the presence of subacromial spurs and of the width of the subacromial space; (3) we did not address the issue of single versus double-row repair [47,48,49]; (4) we did not image the operated shoulder at the time of functional assessment; (5) we did not evaluate fatty infiltration of the relevant muscles following RC repair.

In conclusion, within the limitations outlined above, better results were obtained by the patients who received a limited early passive motion rehabilitation protocol, in which passive external rotation ROM, as well as active pendulum exercise, were restricted during the first 2 weeks after surgery, compared to patients who underwent aggressive early passive motion rehabilitation.

## 5. Conclusions

In this study, two different rehabilitation protocols after arthroscopic RCR for the management of full-thickness RC tears were compared. Better clinical and functional outcomes were obtained by the patients who underwent restricted rehabilitation, in which passive external rotation ROM, as well as active pendulum exercise, were excluded during the first 2 weeks after surgery, compared to patients who received an aggressive early motion rehabilitation protocol including passive external rotation up to 30° and active pendulum exercises. The difference between the two groups resulted in statistically significant concerning active forward flexion ROM. However, no statistically significant differences were found between the two groups concerning the other ROMs analyzed, clinical scores, and muscle strength.

## Figures and Tables

**Table 1:** Demographic and surgical details of the patients.

Demographic and Surgical Details of the Patients			
	Group A	Group B	*p*-Value
**Male/Female**	23;26	22;29	0.6
**Mean age ± standard deviation**	58.2 ± 10	55.2 ± 8.1	0.2
**Dominant/nondominant**	40;10	44;12	0.8
**Trauma/no trauma**	13	12	0.5
**Medium/large tears**	37;13	45;11	0.1
**Long head biceps tear/tendinopathy**	14;8	25;11	0.1
**Long head biceps tenotomy/tenodesis**	20;7	20;12	0.8
**Mean no. of anchor**	1.71	1.76	0.6
**Follow up**	4.2 ± 2	3.9 ± 1.9	0.4

**Table 2:** Clinical scores.

Clinical Scores			
	Group A	Group B	*p*-value
**UCLA**	27.3 ± 7.5	26 ± 6.7	0.3
**Wolfgang**	13.7 ± 3.3	13.1 ± 2.7	0.3
**Oxford**	22.6 ± 10.7	23.9 ± 11.6	0.5
**UCLA Pain**	7 ± 2.8	6.4 ± 2.7	0.2
**Wolfgang Pain**	2.8 ± 1	2.9 ± 0.8	0.9

**Table 3:** Postoperative ROM (°) and strength (range).

Postoperative ROM and Strength (range)		*p*-value
**Passive Forward flexion**		0.7
**Group A**	171 ± 22.9 (90–180)	
**Group B**	169.9 ± 22 (90–180)	
**Passive External rotation**		0.5
**Group A**	73.6 ± 22.2 (0–90)	
**Group B**	70.7 ± 24.5 (0–90)	
**Passive Internal rotation**		0.9
**Group A**	82.2 ± 17.7 (30–90)	
**Group B**	81.9 ± 18 (10–90)	
**Active Forward flexion**		0.04
**Group A**	167.3 ± 26 (90–180)	
**Group B**	156.5 ± 30.5 (90–180)	
**Active External rotation**		0.9
**Group A**	44.3 ± 18.2 (0–90)	
**Group B**	44.5 ± 18.1 (10–90)	
**Strength Forward flexion**		0.3
**Group A**	45.08 ± 90.16 N (19.6–107.8)	
**Group B**	41.16 ± 20.58 N (9.8–107.8)	
**Strength External rotation**		0.3
**Group A**	46.06 ± 19.6 N (14.7–107.8)	
**Group B**	43.12 ± 18.62 N (16.66–102.9)	
**Strength Internal rotation**		0.5
**Group A**	65.66 ± 27.44 N (9.8–107.8)	
**Group B**	62.72 ± 23.52 N (29.4–107.8)	
